# Salicylic Acid Improved the Growth of *Dunaliella salina* and Increased the Proportion of *9-cis*-β-Carotene Isomers

**DOI:** 10.3390/md23010018

**Published:** 2025-01-01

**Authors:** Shuaicheng Xiang, Xiaoting Qiu, Xiaojun Yan, Roger Ruan, Pengfei Cheng

**Affiliations:** 1 College of Food Science and Engineering, Ningbo University, Ningbo 315211, China; 13586929956@163.com (S.X.); qiuxiaoting@nbu.edu.cn (X.Q.); 2 School of Marine Sciences, Ningbo University, Ningbo 315211, China; yanxiaojun@nbu.edu.cn; 3 Center for Biorefining, Department of Bioproducts and Biosystems Engineering, University of Minnesota-Twin Cities, Saint Paul, MN 55108, USA

**Keywords:** *Dunaliella salina*, salicylic acid, β–carotene, *9-cis* isomer, salt tolerance

## Abstract

*Dunaliella salina* is an important source of natural β-carotene (containing *9-cis* and *all trans* isomers) for industrial production. The phytohormone salicylic acid (SA) has been proven to have impacts on the stress resistance of higher plants, but research on microalgae is currently unclear. In this study, the effects of SA on the growth, biochemical composition, antioxidant enzyme activity, key enzymes of β-carotene synthesis, and cis-and trans-isomers of β-carotene in *D. salina* under different salt concentrations were investigated. The results were shown that at concentrations of 1.5, 2, and 2.5 M NaCl, the antioxidant enzyme activity and key enzymes for β-carotene synthesis in algal cells were significantly increased, but the content and proportion of *9-cis* isomer in β-carotene isomers decreased. The addition of SA significantly increased the growth and antioxidant enzyme (SOD, MDA) activity, as well as the synthesis of key enzyme phytoene synthase (PSY), phytoene desaturase (PDS), and lycopene β cyclase (LCYB) of *D. salina* under high-salinity conditions. It is worth noting that under the treatment of SA, the proportion of *9-cis* isomer in the three salt concentrations (1.5, 2, 2.5 M NaCl) significantly increased by 32.09%, 20.30%, and 11.32%, respectively. Moreover, SA can not only improve the salt tolerance of *D. salina*, but also increase the proportion of *9-cis* isomer, with higher physiological activity in β-carotene, thereby enhancing the application value of *D. salina*.

## 1. Introduction

Carotenoids are a class of naturally occurring fat-soluble pigments, mainly terpenoids, consisting of eight isoprenoid units linked at the head and tail which are commonly found in higher plants, microorganisms, algae, and a few animals [1,2]. β-carotene, an important member of the carotenoid family, is abundant in plants and fruits, and is considered to be an important source of vitamin A in the human diet [3]. β-carotene is widely used in the food, nutrition, feed, and pharmaceutical industries due to its antioxidant capacity, immune enhancement, heart protection, and cancer prevention [4]. In addition, some plant extracts can be used as a biological composite material to inhibit the growth of pathogens and are widely used in agriculture [5,6]. β-carotene has a wide range of isomers [7], which can include *all-trans*-β-carotene and *9-cis*-β-carotene [8]. Unlike *all-trans*-β-carotenes, the chemical synthesis of *9-cis*-β-carotene is difficult. The *all-trans* isomer is insoluble in oil and easily crystallizes, whereas *9-cis*-β-carotene has higher oil solubility and does not crystallize easily [9,10]. Notably, the higher the ratio of *9-cis* and *all-trans*-carotenes, the greater the antioxidant and anticancer activity. Most of the β-carotene currently on the market is synthesized (chemically) and the proportion of *9-cis*-β-carotene is small [11,12]. Therefore, natural sources of β-carotene are receiving more and more attention. Moreover, the role of isomerase or light in β-carotene isomerization is also worthy of attention. Recent studies have shown that exposure to red light can significantly increase the content of *cis-*β-carotene in *D. salina* [13].

Microalgae are an important source of natural β-carotene production. *D. salina*, as a single-celled green algae, is recognized as the best source of β-carotene [14,15]. *D. salina* is known for its high tolerance of salinity, mainly in the ocean, salt lakes, and other regions, and is currently one of the most salt-tolerant eukaryotic photosynthetic organisms in the world [16]. *D. salina* also has a high content of *9-cis* isomer and is considered one of the natural sources of *9-cis*-β-carotene [17]. *D. salina* stores large amounts of β-carotene in the form of droplets in the chloroplasts to avoid damage caused by changes in environmental conditions. Therefore, some stress conditions such as N deficiency, low temperature, and strong light induce cells to accumulate β-carotene while causing damage to chloroplasts or algal cells, failing to achieve the desired effect [18]. Therefore, it is of great significance for the development of β-carotene production in *D. salina* to find a method to promote the accumulation of β-carotene without affecting the physiological state of algal cells.

Phytohormones are prevalent in higher plants and play crucial roles as signaling molecules in mediating plant cell growth, development, and environmental stresses. Salicylic acid (SA) is an endogenous phytohormone with a simple phenolic structure that plays a key role in the regulation of physiological processes [19]. SA has been shown to improve the growth of microalgae [20], affect microalgal cellular nitrogen conductance, and influence intracellular transformations of microalgae in additive, synergistic, and antagonistic ways [21]. It has been reported that SA is involved in the signal regulation of astaxanthin synthesis in *Haematococcus pluvialis*. An appropriate concentration of SA can increase the expression of the carotene gene in *H. pluvialis* [21] and further improve the astaxanthin accumulation and antioxidant activity [22,23]. Astaxanthin and β-carotenoids are both physiologically produced from the basic isoprene molecule, and have a 40-carbon unsaturated skeleton. Astaxanthin is a pigment belonging to the lutein family and an oxygenated derivative of carotenoids, which have a similar chemical structure to β-carotene [24]. However, the effect of SA on the synthesis of β-carotene, and its isomers in *D. salina*, is not clear.

Based on these issues, this paper investigated the effects of the exogenous phytohormone SA on the growth biochemistry, antioxidant activity, and key enzymes and isoforms of β-carotene synthesis in *D. salina*. The aim of this study was to determine the effect of SA on the growth and β-carotene accumulation of *D. salina*, and provide a theoretical basis for the production of *9-cis*-β-carotene by *D. salina*.

## 2. Results

### 2.1. Effects of Phytohormone on the Growth of D. salina

**Figure 1:**
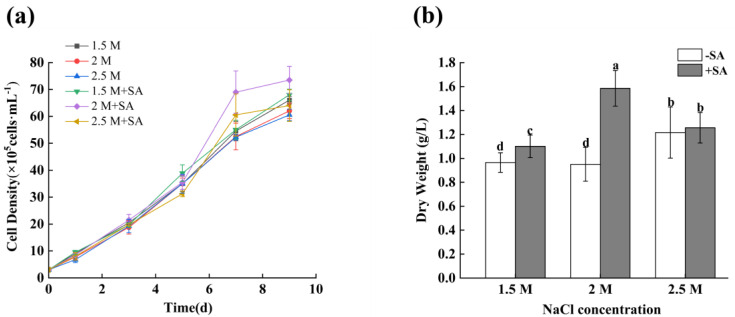
The effects of salinity and SA on the growth of *D. salina*. (**a**) cell density, (**b**) dry weight. Note: values are mean ± SD, obtained from three independent groups. Significant differences between values were calculated at *p* < 0.05 using a Tukey test and marked by different letters; “a–d” indicates a significant difference.

The effects of phytohormone SA on the growth of *D. salina* were investigated at different salinities of 1.5 M, 2 M, and 2.5 M. When SA was not added, it can be seen from Figure 1a that the density of algal cells decreased with the increase in salinity concentrations, and the density of algal cells was relatively high under the control group of 1.5 M salinity. After 9 days of culture at 1.5 M, 2 M, and 2.5 M, the cell densities were 6.60 × 10^6^ cell/mL, 6.20 × 10^6^ cell/mL and 6.05 × 10^6^ cell/mL, respectively. When 1 mM SA was added, the cell density of *D. salina* increased under three salinity conditions, and the cell density was 7.35 × 10^6^ cell/mL at 2 M salinity. Figure 1b shows that there was no significant change in the biomass of *D. salina* under three salinity conditions. When 1 mM SA was added, the biomass of *D. salina* increased under three salinity conditions, and the highest biomass was 1.59 g/L at 9 days under the conditions of SA combined with 2 M salinity.

### 2.2. Effects of Phytohormone on Biochemical Components of D. salina

**Figure 2:**
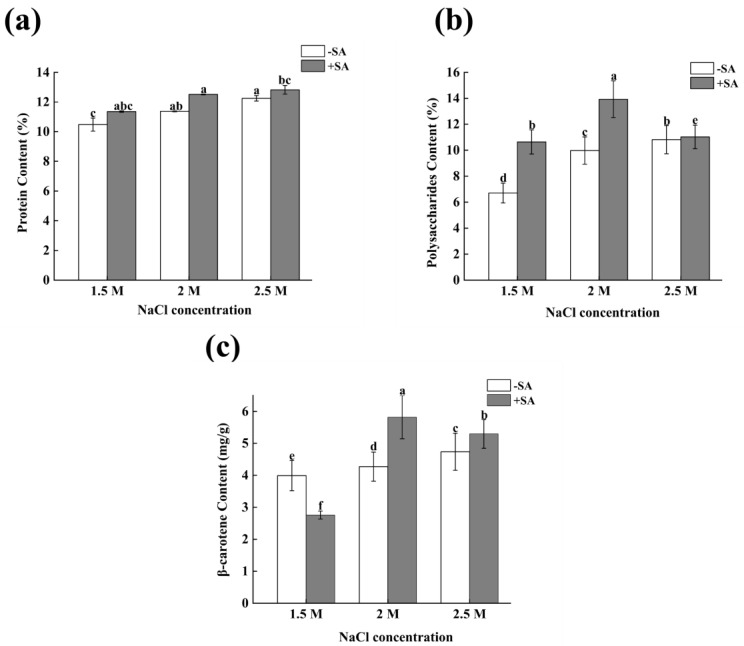
The effects of salinity and SA on the biochemical components of *D. salina*. (**a**) the content of protein, (**b**) the content of polysaccharides, (**c**) the content of β-carotene. Note: values are mean ± SD, obtained from three independent groups. Significant differences between values were calculated at *p* < 0.05 using a Tukey test and marked by different letters; “a–f” indicates a significant difference.

It can be seen from Figure 2a that the protein content increased with the increase in salinity under the three salinity conditions. Compared with the control group (1.5 M), the proteins increased by 8.50% and 17.00% at the salinity of 2 M and 2.5 M, respectively, and there was a significant difference (*p* < 0.05). After adding SA, the protein content of each treatment group improved, increasing by 8.38%, 19.55%, and 22.52%, respectively, under 1.5 M, 2 M, and 2.5 M salinity conditions, and there was a significant difference (*p* < 0.05). The protein content was the highest at 2.5 M salinity with SA, with a content of 12.83%.

From Figure 2b, it can be seen that the content of polysaccharide in *D. salina* increased with the increase in salinity, without phytohormone SA. Compared with the control group (1.5 M), the polysaccharide content under 2 M and 2.5 M salinity reached 9.97% and 10.81%, respectively, which was an increase of 48.84% and 61.34%, respectively; both had significant differences (*p* < 0.05). After the addition of SA, the polysaccharide content was relatively high under the conditions of 2 M salinity, which was 13.93%, but at 2.5 M salinity, the polysaccharide content was significantly reduced to only 5.02% (*p* < 0.05).

It can be seen from Figure 2c that the content of β-carotene in *D. salina* increased with an increase in salinity, without SA. Compared with the control group (1.5 M), the content of β-carotene increased by 7.00% and 18.66% under 2 M and 2.5 M salinity, respectively, with significant differences (*p* < 0.05). After adding SA, under normal salinity conditions (1.5 M), the content of β-carotene was lower than that of the control group without SA, and the content decreased by 30.87% (*p* < 0.05). However, under the conditions of 2 M and 2.5 M salinity, the content of β-carotene after adding SA was higher than the corresponding values without SA, which increased by 45.87% and 32.72%, respectively, with significant differences (*p* < 0.05).

### 2.3. Effects of Phytohormone on the Antioxidant Activity of D. salina in Response to Salinity Changes

**Figure 3:**
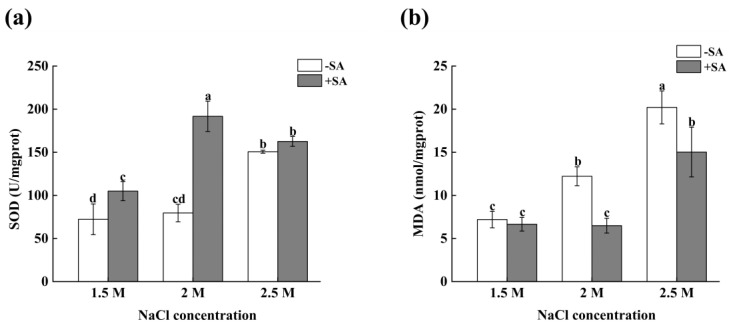
The effects of phytohormones on the antioxidant activity of *D. salina* in response to salinity changes. (**a**) SOD, (**b**) MDA. Values are mean ± SD, obtained from three independent measurements. Significant differences between values were calculated at *p* < 0.05 using a Tukey test and marked by different letters; “a–d” indicates a significant difference.

From Figure 3a, it can be seen that the superoxide dismutase (SOD) increased with the increase in salinity without adding SA. The SOD was the highest (150.69 U/mgprot) at 2.5 M salinity, which was higher than that at 2 M salinity and normal salinity (1.5 M), respectively. After adding SA, the SOD of the experimental groups, with SA added at 1.5 M, 2 M, and 2.5 M salinity, increased by 45.23%, 165.36%, and 124.98%, respectively, compared with those without SA (*p* < 0.05).

It can be seen from Figure 3b that the malondialdehyde (MDA) increased significantly with the increase in salinity under three salinity conditions. The MDA was the highest at 2.5 M salinity (20.21 nmol/mgprot). Compared with normal salinity (1.5 M), the MDA at 2 M salinity increased by 69.74% (*p* < 0.05). Compared with the normal salinity (1.5 M), the MDA at 2.5 M salinity increased by 180.81%, and there was a significant difference (*p* < 0.05). The MDA of the experimental group with SA under 1.5 M and 2 M salinity conditions decreased when compared with the corresponding values without SA. Under 1.5 M and 2 M conditions, the MDA decreased by 7.53% and 9.82%, respectively.

### 2.4. Effects of Phytohormones on Key Enzymes in β-Carotene Synthesis of D. salina

**Figure 4:**
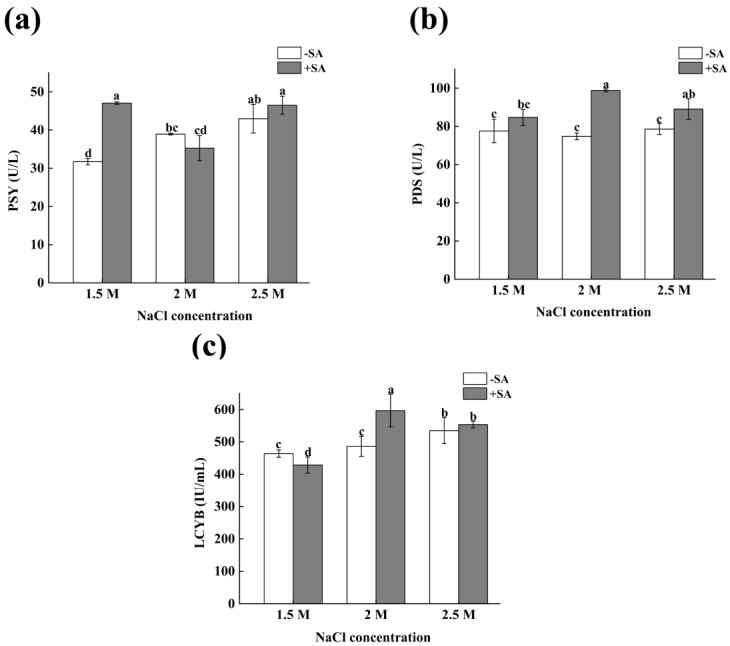
The effects of phytohormones on key enzymes in the β-carotene synthesis of *D. salina*. (**a**) PSY, (**b**) PDS, (**c**) LCY-B. Note: values are mean ± SD, obtained from three independent groups. Significant differences between values were calculated at *p* < 0.05 using a Tukey test and marked by different letters; “a–d” indicates a significant difference.

The changes in related enzymes during the synthesis of β-carotene were shown in Figure 4. Without SA, the content of phytoene synthase (PSY) increased significantly with the increase in salinity, and the content of PSY increased by 26.18%, with significant difference under the conditions of 2.5 M salinity. After adding SA, the PSY content increased at 1.5 M and 2.5 M salinity, and the PSY content increased by 32.58% at 1.5 M salinity, with a significant difference. In the absence of SA, the content of phytoene dehydrogenase (PDS) did not change significantly at the three salinities (1.5 M, 2 M, 2.5 M). After adding SA, the PDS content was significantly increased, and the highest was 98.83 U/L at 2 M salinity, which was 27.37% higher than that of the control group (*p* < 0.05). Without SA, the content of lycopene β-cyclase (LCYB) increased with the increase in salinity. Compared with normal salinity (1.5 M), LCYB content increased by 15.38% (*p* < 0.05) at 2.5 M salinity. The content of LCYB decreased under normal salinity conditions with addition of SA, which may be related to the inhibition of β-carotene synthesis under low-salinity stress. However, under 2 M and 2.5 M salinity conditions, the content of LCYB increased significantly (*p* < 0.05), increasing by 28.79% and 19.34%, respectively. The above results indicate that SA can significantly increase the content of key enzymes in the β-carotene synthesis pathway, thereby promoting the accumulation of β-carotene.

### 2.5. Effects of Phytohormones on the Structure of β-Carotene in D. salina

**Figure 5:**
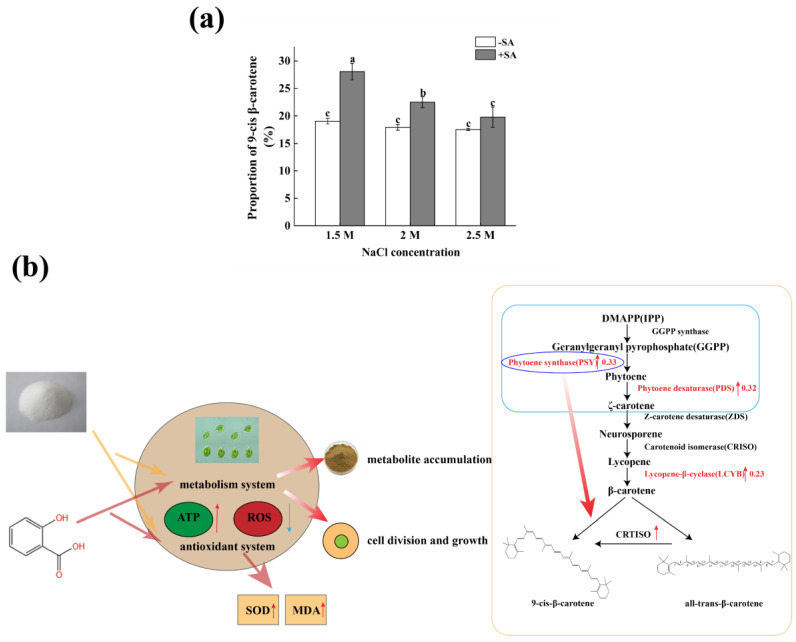
(**a**) The effects of salinity and SA on the growth and β-carotene structure of *D. salina*; (**b**) the possible mechanism of salinity and SA affected the cell growth and β-carotene synthesis of *D. salina*. Note: values are mean ± SD, obtained from three independent group. Significant differences between values were calculated at *p* < 0.05 using a Tukey test and marked by different letters; “a–c” indicates a significant difference. Red arrow represents up-regulated; blue arrow represents down-regulation.

The natural β-carotene synthesized by *D. salina* is a mixture of *cis* and *all-trans* isomers. The ratio of these two isomers was affected by environmental conditions, such as temperature, radiation, and salinity. Since *all-trans*-β-carotene is easy to crystallize, and the *9-cis* structure does not form crystals, the relative ratio of the two isomers will also change during extraction and post-processing [25]. It can be seen from Figure 5a that there was no significant change in the proportion of *9-cis* isomers under the three salinity conditions without SA addition. When the phytohormone SA was added, the proportion of *9-cis* isomers under various salinity conditions (1.5, 2, 2.5 M) increased significantly, 32.09%, 20.30%, and 11.32% higher than the corresponding values without SA, respectively (*p* < 0.05). This may indicate that phytohormone SA may increase the proportion of *9-cis* isomers in β-carotene of *D. salina*.

## 3. Discussions

Previous studies have shown that when the salinity of the growth environment is too high, the internal osmotic pressure of *D. salina* rises rapidly, but the regulation of osmotic pressure requires a lot of energy, so high-salinity conditions will lead to the slow growth of *D. salina* [26,27]. SA, as an endogenous phytohormone, plays a key role in the regulation of many physiological processes and can promote the growth and metabolism of microalgae [18]. It has been found that SA can promote the ATP level in the cells of green alga *H. pluvialis* and promote the growth of *H. pluvialis* [28]. In addition, the use of SA can promote the growth and biomass accumulation of *A. platensis* [29]. Fu et al. [30] found that SA has a positive effect on carbon assimilation, DNA replication, and TCA cycle, and can be used as a new signal molecule to accelerate the growth and proliferation of microalgae. Mirshekari et al. [31] found that under nitrogen deficiency conditions, SA can establish an enzyme balance to regulate metabolite levels and guide them into the growth process, thereby enhancing adaptation to nitrogen starvation. This may be one of the important reasons why phytohormone SA alleviates the tolerance of *D. salina* to high salt. However, this specific mechanism also needs to be further studied.

The active functional substances in microalgae, such as polysaccharides, proteins, and pigments, have many functions such as anti-oxidation, anti-inflammation, and anti-tumor properties, as well as immunity improvement [32]. The accumulation of protein content in *D. salina* cells under salt stress can be attributed to higher protein turnover levels. Changes in protein turnover in *D. salina* cells and other higher plants under stress conditions have been reported [33,34]. Protein turnover is a cellular phenomenon that regulates protein balance during stress, such as the homeostasis of amino acids, the rearrangement of proteins, and the establishment of enzyme balance. These changes are the mechanisms for the establishment of protein balance under salinity stress. The accumulation of polysaccharide content in *D. salina* under salt stress can be attributed to the increase in chlorophyll content in *D. salina*. The carbon flux between starch production in chloroplasts, glycerol synthesis in cytoplasm, and carotenoid accumulation are some important physiological responses under stress conditions [35]. Farkas et al. [36] found that when algae grew under different salinity stress, a slight increase in polysaccharide content was observed. In addition, under salt stress, due to the response to stress conditions, a large amount of carbon and ATP was directed from the synthesis of energy compounds, used for growth in the synthesis of metabolite carotenoids, and the content of β-carotene significantly increased. This is similar to the results of this study. When the salinity content increased to 2.5 M, the protein and polysaccharide content of *D. salina* increased, and the content of β-carotene also increased. It was found that the difference in salinity concentration could increase the content of β-carotene in *D. salina*. When the salt concentration was 30 ppt, the content of β-carotene reached 2.312 mg/L [37].

The results showed that SA had a significant effect on the content of protein and polysaccharide in *D. salina*. Our strain was similar to other research results. SA promoted the growth and metabolism of organisms in plants, which may lead to temporary low-level oxidative stress in plants, thus improving the antioxidant capacity of plants by inducing the synthesis of protective compounds [35]. Proteins and carbohydrates (polysaccharides) are involved in various stress responses of plants [38], which may be related to the increase in proteins and polysaccharides in *D. salina*. Studies also have shown that SA can significantly increase the accumulation of polysaccharides in *Nostoc flagelliforme*, an *increase of more* than 20% [39]. However, under low salt stress, the addition of SA reduced the content of polysaccharides and β-carotene in *D. salina*, which may be related to oxygen consumption and ROS production. The decrease in ROS production and stress intensity may lead to a decrease in polysaccharide and β-carotene content [40]. In addition, in a previous study, it was found that the addition of exogenous SA can effectively promote the production of astaxanthin in heterotrophic *Chlorella zofingiensis* [41].

As a protective enzyme, SOD can scavenge excessive reactive oxygen species in plants and participate in the physiological and biochemical reactions of organisms to resist various environmental stresses [33]. Under salt stress, microalgae cells accumulate a large amount of reactive oxygen species, and SA can be used as a plant signal transduction hormone to remove reactive oxygen species through antioxidant action. This can enhance the antioxidant capacity of plants by activating their antioxidant system [42]. During the growth of algae cells, the production of MDA is considered to be an indicator of membrane lipid peroxidation damage, which can reflect the antioxidant capacity and damage degree of plants [43]. With the increase in salinity, the MDA value increased, indicating that high salinity may cause damage to the cell membrane. The MDA value decreased, which may be related to the alleviation of oxidative stress in microalgae cells by SA, thereby reducing membrane lipid peroxidation [44]. SA is a phenolic derivative that induces cell responses to biotic and abiotic stresses. SA is also a key information molecule that regulates stress response and can improve antioxidant capacity by activating antioxidant enzyme activity and inducing the genes responsible for protective mechanisms [45]. Therefore, exogenous SA can regulate the signal network of microalgae and enhance the stress resistance, so as to maintain cell homeostasis. In this study, under salinity stress, the addition of SA increased the activity of SOD in *D. salina*, and significantly reduced the content of MDA, which alleviated the oxidative damage of *D. salina* caused by salinity.

The current research has not yet clarified how *9-cis*-β-carotene is formed in *D. salina*. A previous study showed that the key enzyme in the accumulation of β-carotene in microalgae may be related to the role of phytoene synthase (PSY), which is a key regulator of the biosynthesis of *D. salina* isomers (*trans and cis*) [46]. Darvidi et al. [47] reported that enhancing the expression of the phytoene synthase (PSY) gene can affect the conversion of *all-trans*- to *9-cis*-β-carotene. Kouidhi et al. [48] found that the use of chimeric peptides can increase the yield of β-carotene in the process of extracting β-carotene from *D. salina*. The interaction energy of the two chimeric peptides on cis- and trans-β-carotene is not the same. Chimeric PP2 seems to have the same interaction with these two isomers. However, the chimeric PP3 seems to be more specific to the cis-β-carotene form. Notably, Alder et al. [49] found a protein that catalyzes the isomerization of all-trans to *9-cis*-β-carotene, which was initially identified as a part of the strigolactone biosynthesis pathway in rice, known as D27. Therefore, it is speculated that exogenous SA can not only increase the biosynthesis rate of β-carotene in *D. salina* and reduce the formation rate of reactive oxygen species (ROS) by up-regulating the phytoene synthase, but also convert the existing *all-trans*-β-carotene into *9-cis*-β-carotene by up-regulating the β-carotene isomerase [50]. In this study, the addition of SA significantly enhanced the content of PSY, increased the biosynthesis of β-carotene and reduced the formation rate of ROS, and catalyzed the isomerization of *all-trans* to *9-cis* (Figure 5b). But as salinity increases, the proportion of *9-cis* isomer decreases, which may be due to microalgae being able to accumulate more β-carotene under stress conditions. Under low salt stress conditions, *D. salina* upregulates the gene for lycopene synthase, which affects the conversion of all-trans- to *9-cis*-β-carotene. Therefore, the proportion of *9-cis*-β-carotene is relatively high under low-salinity conditions. The addition of SA may also increase the concentration of β-carotene isomerase and catalyze the conversion of *all-trans*-β-carotene, which may be related to the relevant genes *PSY*, *PDS*, and *LYCB* in the process of β-carotene synthesis, but the specific mechanism needs further study.

## 4. Materials and Methods

### 4.1. Algal Strains

The microalgae strain *D. salina* FACHB-815 was purchased from the Institute of Aquatic Biology, Chinese Academy of Sciences.

### 4.2. Culture Condition

*D*. *salina* was preserved and cultured using the Dunaliella medium, and the formula for this medium was provided by the Freshwater Algae Culture Collection at the Institute of Hydrobiology (FACHB) (Table 1 and Table 2). The salt concentration of the medium was changed by adjusting the NaCl content; the pH was adjusted to about 7.5 with 0.1 mol/L HCl, and the preparation was completed by autoclave sterilization (121 °C, 20 min) and then cooled down for use. The cultivation conditions were as follows: the activated logarithmic growth-stage *D. salina* was taken and inoculated with an initial inoculum density of 3 × 10^5^ cells/mL in 1 L conical flasks with a culture volume of 600 mL. *D. salina* was cultivated in an illuminating incubator (GXM-508BM, Ningbo Jiangnan Instrument Factory, Ningbo, China) by using a cold white fluorescent lamp with an illumination intensity of 3000 ± 100 lx. The temperature was set at 25 ± 2 °C, and the photoperiod was 12 h: 12 h (light–dark). The carbon source in this research was just air (0.038% CO_2_) at 1 vvm flow rate. The salt concentrations were set at 1.5, 2, and 2.5 M NaCl, where the 1.5 M NaCl group was the control group. Salt-adapted algal strains were inoculated in the above media at different salt concentrations, and the media at each concentration was divided into two parts. One part was treated with 1 mM SA (the data are shown in the Supplementary Materials), and the other part was not treated. Both groups were activated multiple times under the above culture conditions.

### 4.3. Growth and Biochemical Measurements

#### 4.3.1. Determination of Algal Cell Number and Biomass

We placed 1 mL of algal cells into 2 mL eppendorf (EP) tubes (sampling time: 0 day, 1 day, 3 day, 5 day, 7 day, 9 day). Before sampling, each sample was shaken clockwise and counterclockwise for 10 times. After sampling, we added 10 μL of Lugol’s iodine solution to the EP tube and the microalgal cells were fixed by shaking. The 10 μL sample was shaken and placed in a blood cell counting plate, and the algal cell density was observed and counted under a microscope.

The biomass of *D. salina* was measured by the dry weight method [51]. In detail, 10 mL of algae solution was taken at a specific time, and it was filtered onto the weighed 0.45 μm acetate-fiber filter membrane using a suction filter device. The samples were dried in a drying oven at 85 °C for 4 h, and then cooled at room temperature. The weight was measured by using an analytical balance.

#### 4.3.2. Determination of Polysaccharide Content

In this study, the polysaccharide content was determined by the phenol-sulfuric acid method [52]. The polysaccharide was extracted with hot water, ultrasonication, complex enzyme, and other methods, and the phenol solution and concentrated sulfuric acid were used for color reaction. The total polysaccharide content was calculated according to the absorbance at 490 nm.

#### 4.3.3. Determination of Protein Content

The protein content of the microalgae was determined by the Coomassie brilliant blue G250 method [53]. The protein extraction solution was used to extract the microalgae powder. After extraction and centrifugation (6000× *g*, 4 °C, 15 min), Coomassie brilliant blue was added to the supernatant, and its absorbance was measured at a wavelength of 595 nm after standing for 2 min. Finally, the corresponding concentration was calculated according to the standard curve.

### 4.4. Determination of Antioxidant Activity of D. salina

#### 4.4.1. Preparation of Crude Enzyme Solution

We took 50 mL of algal cells from each group cultured until the 11th day; these underwent centrifugation at 4 °C with 6000× *g* for 10 min. The supernatant was collected, and the algae cells were washed with phosphate buffer solution (PBS) at pH 7.6 2–3 times. Then, the same amount of PBS was added, and the cells were disrupted using a homogenizer (MP Biomedical, FastPrep-24 5G). The homogenization procedure was as follows: homogenized for 30 s, suspended for 5 s, 3 cycles in 1 cycle, for a total of 15 rounds; then, 6000× *g* centrifugal supernatant was used at 4 °C. The supernatant was incubated in a water bath at 37 °C for 30 min, and then centrifuged at 8000× *g* at 4 °C for 10 min. The supernatant was taken as the crude enzyme solution [54].

#### 4.4.2. Determination of Antioxidant Enzyme Activities

The activity of superoxide dismutase and the content of malondialdehyde in algal cells were determined by using SOD and MDA kits (A001-3, Nanjing Jiancheng Institute of Biological Engineering, Nanjing, China), respectively. A certain number of algae cells were taken out from the experimental group and the control group, centrifuged at 6000× *g* and 4 °C for 10 min, and the supernatant was removed. The algae cells were washed 2–3 times with phosphate buffer solution (PBS) at pH 7.6, and then the same amount of PBS was added. The cells were broken by homogenizer, and the supernatant was centrifuged at 6000× *g* and set at 4 °C. The supernatant was incubated in a water bath at 37 °C for 30 min, and then centrifuged at 8000× *g* and 4 °C for 10 min. The supernatant was taken as the crude enzyme solution. After configuring the substrate application solution and enzyme working solution, the crude enzyme solution was diluted with different concentrations and mixed. The absorbance value was measured using a microplate reader, and the SOD and MDA activities were calculated after incubation at 37 °C for 20 min [55].

### 4.5. The Determination of the Content of Key Enzymes of the β-Carotene Synthesis Pathway and Their Isomers

#### 4.5.1. The Determination of Key Enzymes in the β-Carotene Synthesis Pathway

The activities of phytoene synthase (PSY), phytoene dehydrogenase (PDS), and phytoene β-cyclase (LCYB) were determined by phytoene synthase (PSY) ELISA kit, phytoene dehydrogenase (PDS) and phytoene β-cyclase (LCYB) ELISA kit (MK30247B, Jiangsu Sumeike Biotechnology Co., Ltd., Zhenjiang, China), respectively. After the sample was incubated and washed, the sample was colored with the enzyme substrate TMB, changing the color of the sample to blue under the catalysis of peroxidase. Under the action of acid, it was converted into the final yellow color. The depth of color was positively correlated with PSY, PDS, and LCYB in the sample.

#### 4.5.2. Determination of β-Carotene Isomer Content

In this research, β-carotene was extracted using a pigment extraction solvent (methanol–dichloromethane = 1:1, *v*/*v*). Each centrifuge tube was labeled and weighed before the sample was collected and frozen in a centrifuge tube. The dried sample was collected and weighed again. The difference between the two was the net weight of the dried sample. We added 500 μL of pigment extract to a 1.5 mL centrifuge tube containing the dried sample, mixed in a small suspension instrument for 30 s, which was shaken in an ultrasonic cleaning instrument for 5 min, and the sample was taken out and extracted at −20 °C. After 20 min, the sample was taken out and then centrifuged at 12,000× *g* for 5 min. The supernatant was transferred to a new centrifuge tube (1.5 mL). The above steps were repeated until the supernatant was almost colorless, and we then started the sample-loading and filtration operation. The extraction solution in the 1.5 mL centrifuge tube containing the supernatant was added to 1.5 mL to complete the constant volume step, and then about 1 mL of the pigment-containing extraction solution was extracted using a syringe. After replacing the needle with a filter, the pigment sample was injected into the HPLC sample bottle, and the cap was tightened and tested on the machine. The pigment content was determined by using Thermo ScientificTM DionexTM UltiMateTM 3000 liquid chromatograph (ThermoFisher Scientific, Shanghai, China), and light was avoided throughout the experiment.

### 4.6. Data Statistics and Analysis

The experiment was set up as three independent biological parallel groups. Through single-factor and multi-factor analysis of variance, the statistical differences between the experimental groups were obtained. *p* < 0.05 indicated that there was a significant difference between the groups, and there was statistical significance. Statistics 26 software was used to analyze the experimental results.

## 5. Conclusions

This study investigated the effects of phytohormone SA on the growth, biochemical components, antioxidant enzyme activity, key β-carotene synthesis enzymes, and isomers of *D. salina* under different salt concentrations. The results showed that SA could improve the growth of *D. salina* and enhance its biochemical composition and antioxidant enzyme activity under salt stress. The protein, polysaccharide, and β-carotene contents of *D. salina* increased by 22.52%, 13.93%, and 45.87%, respectively. In addition, the addition of SA increased the key enzyme PSY for β-carotene synthesis by 32.58%, and the proportion of *9*-*cis*-β-carotene increased from 19.05% to 28.05%. Overall, this research provides a theoretical foundation for the application potential of β-carotene in *D. salina*.

## Figures and Tables

**Table 1:** The medium formula for *D. salina*.

#	Composition	Concentration
1	NaCl	87.69 g/L
2	NaNO_3_	0.42 g/L
3	NaH_2_PO_4_·2H_2_O	0.0156 g/L
4	CaCl_2_·2H_2_O	0.044 g/L
5	KCl	0.074 g/L
6	MgSO_4_·7H_2_O	1.23 g/L
7	NaHCO_3_	0.84 g/L
8	Ferric citrate (1%)	0.5 mL
9	A5 (trace element solution)	1 mL/L

**Table 2:** A5 (trace element solution).

#	Composition	Concentration
1	H_3_BO_3_	2.86 g/L
2	MnCl_2_·4H_2_O	1.86 g/L
3	ZnSO_4_·7H_2_O	0.22 g/L
4	Na_2_MoO_4_·2H_2_O	0.39 g/L
5	CuSO_4_·5H_2_O	0.08 g/L
6	Co(NO_3_)_2_·6H_2_O	0.05 g/L

## Data Availability

The original data are available from the correspondent author on request.

## Supplementary Materials

The following are available online at https://www.mdpi.com/article/10.3390/md23010018/s1, Figure S1: The effect of different concentrations of SA on the growth of *D. salina*.
