# Supplementary material for: Salicylic Acid Improved the Growth of Dunaliella salina and Increased the Proportion of 9-cis-β-Carotene Isomers
Source: Mar Drugs. 2025 Jan 1;23(1):18. doi: 10.3390/md23010018 (PMC11766574; doi:10.3390/md23010018)
Supplement: Supplementary file 1 [file marinedrugs-23-00018-s001.zip › marinedrugs-3371145-supplementary.pdf]

Supplementary material

Supplementary Figure

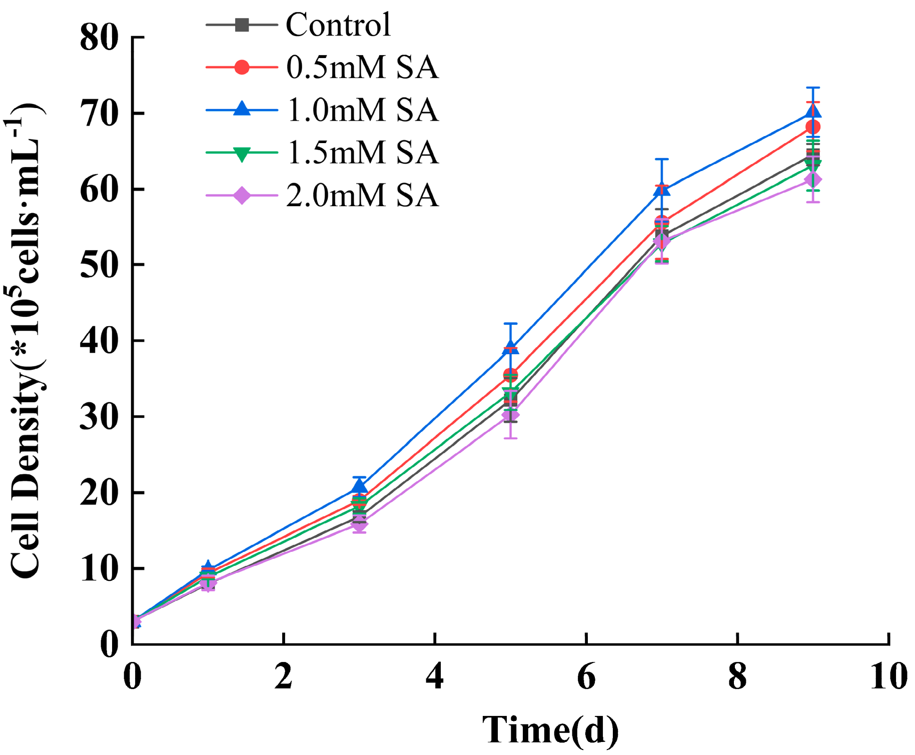

Figure S1. The effect of different concentrations of SA on the growth of *D. salina*.
